# Performance Comparison of Cross- and Forward-Flow Configurations for Multiple-Effect Vacuum Membrane Distillation

**DOI:** 10.3390/membranes12050495

**Published:** 2022-04-30

**Authors:** Abdullah Najib, Hany Al-Ansary, Jamel Orfi, Emad Ali, Fahad Awjah Almehmadi

**Affiliations:** 1 Mechanical Engineering Department, King Saud University, P.O. Box 800, Riyadh 11421, Saudi Arabia; anmohammed@ksu.edu.sa (A.N.); hansary@ksu.edu.sa (H.A.-A.); orfij@ksu.edu.sa (J.O.); 2 Chemical Engineering Department, King Saud University, P.O. Box 800, Riyadh 11421, Saudi Arabia; 3 Department of Applied Mechanical Engineering, College of Applied Engineering, Muzahimiyah Branch, King Saud University, P.O. Box 800, Riyadh 11421, Saudi Arabia; falmehmadi@ksu.edu.sa

**Keywords:** multi-effect vacuum membrane distillation, heat pump system, cross-flow configuration, forward-flow configuration, performance indicators

## Abstract

This work addresses retrofitting the infrastructure of multiple-effect vacuum membrane distillation (V-MEMD) units by using cross-flow configuration (CFC). In this configuration, the feed water is evenly divided and distributed over the effects. In this case, the feed water stream for each effect is kept at a high temperature and low flow rate. This will lead to an increase in the vapor pressure gradient across the hydrophobic membrane and can also maintain the thermal energy of the stream inside the individual effect. It is found that CFC improves internal and global performance indicators of productivity, energy, and exergy. A mathematical model was used to investigate the performance of such a modification as compared to the forward-flow configuration (FFC). The cross-flow configuration led to a clear improvement in the internal performance indicators of the V-MEMD unit, where specifically the mass flux, recovery ratio, gain output ratio, and heat recovery factor were increased by 2 to 3 folds. Moreover, all the global performance indicators were also enhanced by almost 2 folds, except for the performance indicators related to the heat pump, which is used to cool the cold water during the operation of the V-MEMD unit. For the heat pump system, the specific electrical energy consumption, SEEC, and the exergy destruction percentage, *Ψ_des_*, under the best-operating conditions, were inferior when the feed water flow was less than 159 L/h. This can be attributed to the fact that the heat rejected from the heat pump system is not fully harnessed.

## 1. Introduction

Membrane distillation (MD) is an encouraging desalination method to produce high-quality potable water. This technology’s novelty is the combination of the thermally based desalination process and the membrane filtration process in one unit, denoted as trans-membrane evaporation [1,2,3]. Thermal energy is used to heat up saline water to produce water vapor. A micro-porous hydrophobic membrane allows only vapor to pass and separates the pure water vapor from the heated saline water, unlike reverse osmosis technology driven by total pressure. Water separation from salty solution in the MD process depends on the vapor pressure difference. This difference is created by the negative pressure on the permeate side (vacuum pressure in each effect) and feed temperatures at the hydrophobic membrane interface. There are several popular configurations of the MD process, such as the direct contact membrane distillation (DCMD) [3,4], air gap membrane distillation (AGMD) [4], vacuum membrane distillation (VMD) [5,6,7], and sweeping gas MD (SGMD) [4]. The range of pore size of the hydrophobic membranes is often between 0.02 and 1.00 μm [8].

Lately, a handful of research has focused on studying how to enhance distillate production and reduce energy use, both thermal and electrical [9,10,11]. Numerous pilot plants were designed and integrated with renewable energy sources [9,10,11,12,13,14,15,16]. Such as solar energy, geothermal energy, and waste energy. However, productivity is still limited, associated with high thermal and electrical energy intake per volume of production [17]. Other practitioners and researchers studied improving membrane distillation efficiency by recycling thermal energy via using multiple-effect MD (MEMD) systems. One of those innovative ideas was increasing the number of effects to extract the largest amount of distillate water, as studied in [10,12,13,14,15,18]. Moreover, among those innovative ideas is preheating the feed stream by outlet streams (i.e., cooling, distillate, and brine stream) as investigated in [10,12,13,14,15,18]. Another pioneering idea is using the heat pump system to preheat the feed stream by leveraging the heat rejected from the heat pump system through the condenser, as explained in [10,19]. Moreover, the development of the efficiency of fabricated membranes is one of the important factors that played a positive role in improving the distillation process, as reported in previous works [20,21].

There is a lack of research concerned with retrofitting the infrastructure of MEMD. This motivated us to study the possibility of applying the cross-flow configuration (CFC) concept. Several previous studies dealt with the concept of retrofitting and its effectiveness on conventional desalination systems [22,23]. At the beginning of the twenty-first century, several commercial companies (e.g., SIDEM company) developed the infrastructure of conventional thermal desalination (i.e., multiple-effect distillation (MED)) systems under various feed configurations; This led to a significant impact on improving the performance of those systems. Interestingly, studies indicated an increase in production capacity from two to eight million gallons per day, as reported in Ben Amer [23]. El Dessouky et al. [22] were among the first researchers to verify the advantages and disadvantages of the various configurations used in conventional desalination systems. The aims of these configurations are numerous, including raising productivity to avoid the accumulated salinity through cascading and obtain benefits from the heat of the brine leaving the effects. Among those configurations used in developing the infrastructure of the conventional desalination systems are as follows; forward, backward, and cross-flow configurations. The three configurations differ in the feed water flow directions in terms of the gaining of thermal energy and evaporation process, as explained in detail [22]. Indeed, the obtained results proved the effectiveness of the cross-flow configuration.

This paper aims to enhance the performance of the multiple effects of vacuum membrane distillation (V-MEMD) by applying the CFC. This will be investigated theoretically by using a mathematical model developed by Najib et al. [24], which in turn evaluates several key performance indicators (KPI). The results are expressed in terms of variation of productivity, recovery ratio, gain output ratio, specific thermal energy consumption, specific electrical energy consumption, specific thermal exergy consumption, exergetic efficiency, and exergy destruction in the V-MEMD system. Additionally, we will assess the effect of CFC on these KPIs. Specifically, its reflections on the V-MEMD system’s performance under best-operating conditions will be assessed.

## 2. Fundamentals of Multiple-Effect Vacuum Membrane Distillation (V-MEMD)

Membrane distillation (MD) is a promising technology in the field of desalination and water treatment and has attracted many researchers and practitioners in academia and industries. Although there are massive numbers of research activities and investigations related to the development of MD with its various configurations and its association with renewable energy sources [17], the technology still faces challenges that hinder its commercialization. The basic single-stage MD structure exhibits many negative aspects, such as its enlarged specific energy use and modest production capacity. Different remedies for these issues have been reported. One example is the recovery and recycling of energy from the permeate stream [25]. Another example suggests using multiple effects [9,10,12,15,19,26]. Following the concept of the conventional multi-effect distillation (MED) process, several effects of MD can be constructed in series in a compact modular structure forming the multi-effect membrane distillation. This module works under a vacuum where thermal energy is recycled from stage to stage. The benefit of this cascaded structure is producing drinkable water with the lowest energy demands.

Recently, the VMD configuration has been gaining attention from researchers and practitioners due to its appealing features, especially when configured as multiple effects. Sustainable driving force throughout the stages can be obtained by enforcing negative pressure (vacuum) below the equilibrium vapor pressure at the permeate side. This vacuum enhances the vapor pressure variation across the hydrophobic membrane, which in turn increases the permeate flux.

### Experimental Device

**Figure 1:**
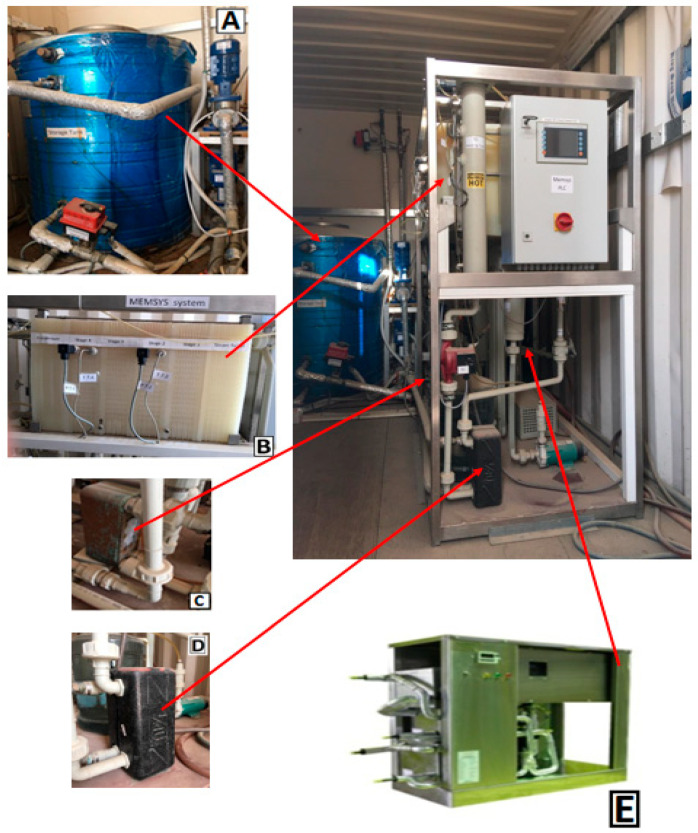
Photographs of the V-MEMD system facilities. (**A**) Thermal storage tank, (**B**) multiple effects, (**C**) HX-2, (**D**) HX-1, and (**E**) heat pump system.

**Figure 2:**
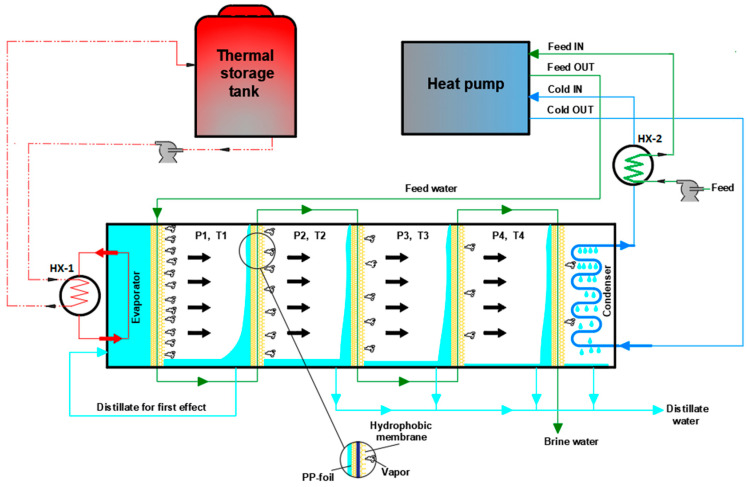
Schematic diagram showing the principle of V-MEMD unit.

The pilot V-MEMD unit, manufactured by MEMSYS (Schwabmünchen, Germany), which operates under automatic control, is governed using a BnR Programmable Logic Controller (PLC). A detailed description of the experimental setup is given in [10,19]. To avoid repetition, a brief description is given here. Figure 1 presents the main parts of the multi-effect membrane distillation under a vacuum unit (V-MEMD). The V-MEMD unit, the thermal storage tank, multiple effects, the second heat exchanger (HX-2), the first heat exchanger (HX-1), and the heat pump system (HP) are shown in Figure 1A–E, respectively. Table 1 lists the main characteristics and specifications of the MD layer. It is important to mention the characteristics and specifications of the MD layer so that they are used to generate experimental data that are used for validating the theoretical models. Figure 2 shows the separation process of the multi-effect membrane distillation under vacuum pressure. One can note three main parts, namely, an evaporator, multiple effects (evaporation-condensation), and a condenser, as described in Figure 1B. The evaporator consists of several membrane frames and is installed in front of the first effect. It is responsible for providing thermal energy to the multi-effect membrane distillation process. A plate heat exchanger (HX-1) is used to transfer heat from the thermal storage tank (i.e., thermal energy source) to the evaporator. In the evaporator, the vapor is produced by evaporating water at low pressure of less than 20 kPa. The latent heat carried by the resulting vapor is transferred to the first effect of multiple effects in order to heat up the feed water in the channels. When the water in the evaporator evaporates, the system automatically compensates for water loss due to evaporation since the system operates at negative pressure. The proposed V-MEMD process contains four consecutive effects (evaporation-condensation). In each effect, the heat of condensation is recovered for the evaporation process at the following effect. The condensation-evaporation process is replicated upwind the effects at constantly declining temperatures. Wherefore, the overall thermal efficiency of the process is governed by the number of effects. For example, adding more effects recovers more energy enhancing the thermal efficiency. Hence, the production of potable water can be magnified. However, this is limited by the ability to maintain enough driving force throughout the effects. It should be reminded that the condenser is constructed of multiple layers made of foil. The function of the condenser chamber is to condense the vapor generated in the terminal effect via the cold water stream. The cold water temperature is maintained constant through the distillation process by the heat pump system. A heat pump system is custom-designed specially made for heating and cooling water. The system features an open frame design for easy servicing and maintenance of the heat exchangers located in it. In addition to being insulated with polyurethane foam to maintain the quality of heat exchange. Controlling the cooling capacity is performed by operating the compressor with an inverter single-phase motor controller that works in conjunction with an analog thermostat. Through the operating cycle, the distilled water produced from the distillation process is cooled by the heat pump’s evaporator while the feed water coming from its source is heated by the heat rejected from the heat pump’s condenser, and this process takes place simultaneously.

## 3. Design Structure and Methodology

**Figure 3:**
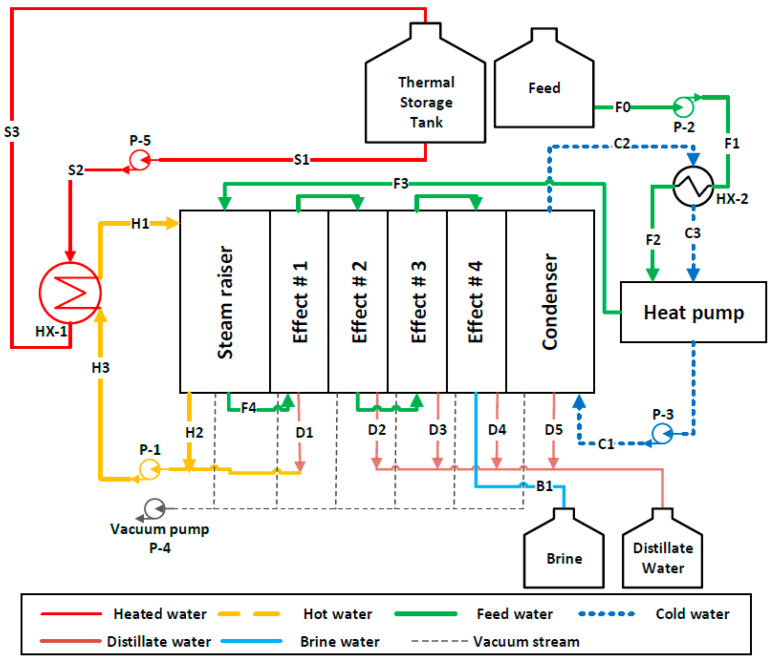
V-MEMD system under forward-flow configuration.

**Figure 4:**
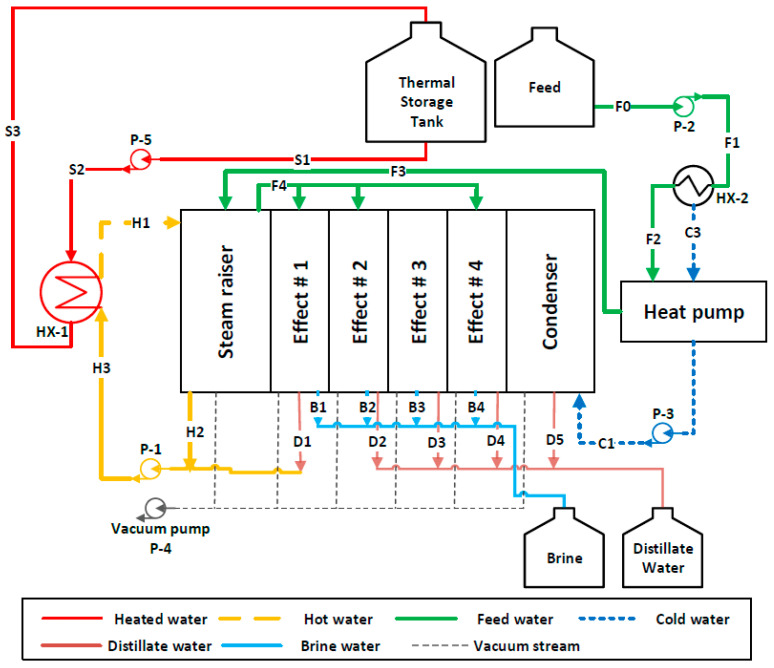
V-MEMD system under cross-flow configuration.

The concept of desalination using MD technology is based on phase change due to applying thermal energy (i.e., heating and cooling energy) as applied to conventional systems (e.g., multi-effects distillation (MED) and multi-stages flash system (MSF)). As shown in Figure 3 and Figure 4, the heated water stored in the thermal storage tank is fed to the V-MEMD unit via an HX-1, which exchanges thermal energy with the hot water stream (*H*1) to raise its temperature to the desired temperature (T*_H_*_1_). Since the V-MEMD unit operates under vacuum pressure, the hot water in the evaporator compartment contributes to heat up the feed water (*F*3) and, at the same time, drives the latent heat to the next effect by the extracted vapor, and then the vapor condenses (*D*1) to reserve a mass of the hot water. The procedure of driving the latent heat is replicated in cascade effects to continuously provide the thermal energy to the feed water that loses its heat as a result of evaporation. Two different configurations of the V-MEMD unit were used in this study; the main difference between them is the way the feed water flows through the cascade effects. Forward-flow configuration (FFC) in experimental works was investigated [10,19] and theoretically studied by Najib et al. [24]. With regard to this configuration, the feed water flows through cascade effects by moving from one effect to the next effect, where circulating water loses a portion of its mass and heat and ends up in brine water (*B*1), as shown in Figure 3. The second is the cross-flow configuration (CFC). The feed water after leaving the evaporator compartment (*F*4) is evenly distributed over all effects. An advantage of this feed-flow pattern is that the feed water enters the effect at a high temperature and low flow rate, which facilitates the compensation of the thermal energy of the feed water in the channels by the thermal energy driven by the previous effect. Each branch of the feed water ends up as brine water (*B*1, *B*2, *B*3, and *B*4), as shown in Figure 4. The cooling energy driven by the heat pump unit effectively contributes to removing the heat accumulated in the condenser compartment. This allows expansion of the thermal energy throughout the cascade effects of the V-MEMD unit (i.e., more productivity). The heat rejected by the heat pump system and HX-2 effectively contributes to minimizing the thermal energy supplied to the V-MEMD unit via preheating the feed water flow (*F*1 and *F*2), as illustrated in Figure 3 and Figure 4. The abbreviations of the V-MEMD components used in Figure 3 and Figure 4 are shown in Table 2.

## 4. Mathematical Model

A schematic diagram of the V-MEMD system is shown in Figure 3 and Figure 4. The system contains the multiple effects vacuum membrane distillation unit (V-MEMD), heat pump system, two heat exchangers (HX-1 and HX-2), and five pumps (P-1 to P-5). In each effect, the V-MEMD unit is modeled through conservation equations and the second law of thermodynamics to evaluate direct outcomes (i.e., masses, temperatures, pressures, and salinities) and properties. In the mathematical model, mass, energy, and entropy generation balance equations have been developed for each major component (i.e., V-MEMD unit, heat pump, and heat exchangers). The following typical assumptions are made:

1. Flow, heat, and mass transfers are supposed to be one-dimensional and steady-state.
2. Kinetic and potential energies of fluids are neglected.
3. Viscous diffusion throughout the membrane is negligible.
4. Each component of the system is rigid and with no fluid leakage.
5. No chemical reactions are involved.
6. Homogeneity and thermal equilibrium of fluids are assumed in both effects and channels.
7. Complete condensation is assumed in each effect.

### 4.1. Mass and Energy Balances

**Figure 5:**
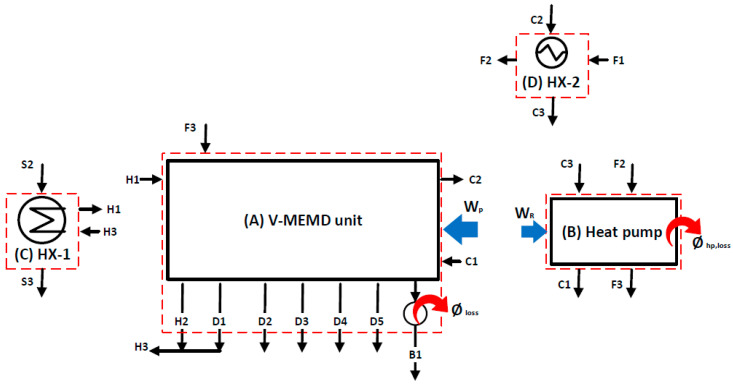
Specification of control volumes for major components: (**A**) V-MEMD unit, (**B**) heat pump system, (**C**) HX-1, and (**D**) HX-2.

Conservation equations are applied to a number of carefully selected control volumes to predict the output results for each major component (i.e., the V-MEMD unit, heat pump, HX-1, and HX-2) of the entire desalination system. These control volumes are described in Figure 5. Indeed, the conservation equations for each control volume were solved simultaneously by successive iterative procedures until overall convergence occurred, as explained by Najib et al. [24].

Having developed the control volumes for each component of the overall process as specified in Figure 5, the conservation law can be written for each control volume as described in the following section.

The control volume (A) for the V-MEMD unit contains a hot water stream, feed water stream, and cold water stream. Conservation equations can be written through this control volume as follows:

(1) $${\dot{m}}_{H1}+{\dot{m}}_{C1}{\dot{+m}}_{F3}={\dot{m}}_{H2}+{\dot{m}}_{C2}+{\dot{m}}_{B1}+\overset{N}{\underset{i=1}{\sum }}{\dot{m}}_{D,i}$$

(2) $$\left[\begin{array}{c} {\dot{m}}_{H1}h_{H1}+{\dot{m}}_{C1}h_{C1}+{\dot{m}}_{F3}h_{F3}+W_{p}={\dot{m}}_{H2}h_{H2}\\ +{\dot{m}}_{C2}h_{C2}+{\dot{m}}_{B1}h_{B1}+\overset{N}{\underset{i=1}{\sum }}{\dot{m}}_{D,i}h_{Df,i}+\emptyset_{loss} \end{array}\right]$$

The heat supplied to the evaporator can be expressed as follows:

(3) $$\emptyset_{H,E}={\dot{m}}_{H1}h_{H1}-{\dot{m}}_{H3}h_{H3}$$

where $\dot{m}$, h, $\emptyset_{loss}$, $\emptyset_{H,E}$, and $W_{p}$ denote mass, enthalpy, heat loss from the brine water stream, heat supplied on the evaporator, and the total work consumed by the V-MEMD unit’s pumps, respectively. H1 and H2 refer to the hot water inlet and outlet, respectively. C1 and C2 refer to the cold water inlet and outlet, respectively. $F_{3}$, $D_{i},$ and B1 refer to the inlet feed water, distillate water streams, and brine water, respectively.

For the control volume (B) comprising the heat pump system, the following mass balance can be written:

(4) $${\dot{m}}_{C3}+{\dot{m}}_{F2}={\dot{m}}_{C1}+{\dot{m}}_{F3}$$

Based on the ideal vapor-compression refrigeration cycle, the cooling heat $\left(\emptyset_{C}\right)$ is absorbed by the evaporator during the phase change of the refrigerant. It is then isentropically compressed to a high pressure by the compressor work $(W_{R}),$ accompanied by a rise in the refrigerant temperature. Therefore, differences between refrigerant temperature and the surrounding temperature result in the following heat rejected $\left(\emptyset_{hp}\right)$:

(5) $$\emptyset_{hp}=\emptyset_{C}+W_{R}$$

Heat rejected $\left(\emptyset_{hp}\right)\;$ from a heat pump system can also be defined as the summation of the heat loss $\left(\emptyset_{hp.loss}\right)$ to the surroundings and the amount of heat used to preheat the feed water.

(6) $$\emptyset_{hp}=\left({\dot{m}}_{F3}h_{F3}-{\dot{m}}_{F2}h_{F2}\right)+\emptyset_{hp,loss}$$

The heat removed from the cold water stream by the heat pump system can be expressed as follows:

(7) $$\emptyset_{C}=\left({\dot{m}}_{C3}h_{C3}-{\dot{m}}_{C1}h_{C1}\right)$$

The work supplied to the heat pump (i.e., refrigeration) system can be predicted by knowing the coefficient of performance COP of the heat pump, which is estimated from its technical description report as 3 (COP = 3).

(8) $$W_{R}=\emptyset_{C}/\mathrm{COP}$$

where the indices F2 and F3 refer to the feed water entering and exiting the heat pump system, respectively. $\emptyset_{hp}$ is the heat rejected, $\emptyset_{c}$ is the heat absorbed by a heat pump system, and $W_{R}$ is the work supplied on the heat pump system.

The control volume (C) refers to the first heat exchanger (HX-1) with the following balance can be developed:

(9) $${\dot{m}}_{S2}+{\dot{m}}_{H3}={\dot{m}}_{S3}+{\dot{m}}_{H1}$$

(10) $${\dot{m}}_{S2}h_{S2}+{\dot{m}}_{H3}h_{H3}={\dot{m}}_{S3}h_{S3}+{\dot{m}}_{H1}h_{H1}$$

The heat supplied to the hot water can also be expressed as follows:

(11) $$\emptyset_{H}={\dot{m}}_{S1}h_{S1}-{\dot{m}}_{S3}h_{S3}$$

The control volume (D) refers to the second heat exchanger (HX-2) with the following balances can be written:

(12) $${\dot{m}}_{F1}+{\dot{m}}_{C2}={\dot{m}}_{F2}+{\dot{m}}_{C3}$$

(13) $${\dot{m}}_{F1}h_{F1}+{\dot{m}}_{C2}h_{C2}={\dot{m}}_{F2}h_{F2}+{\dot{m}}_{C3}h_{C3}$$

### 4.2. Performance Indicators

It is important to evaluate the performance of the V-MEMD system based on specific key performance indicators. The performance indicators used in this study are categorized to assess productivity, energy, and exergy. The following section displays the main performance indicators based on those indicators:

#### 4.2.1. Productivity Indicators

There are performance indicators that are directly related to the amount of water produced from desalination units, especially those that use the membrane distillation technique. Therefore, among those indicators are the permeate mass flux (*J*), recovery ratio (*R*), and concentration factor (*C_f,i_*), as discussed as follows:

One of the most important performance indicators is the permeate mass flux (*J*), which is used to assess the efficiency of the membrane distillation. The overall permeate mass flux of the V-MEMD unit can be expressed as follows [10,24]:

(14) $$J=\frac{\sum_{i=2}^{N}{\dot{m}}_{D,i}}{A}$$

The recovery ratio (*R*) is considered the primary performance indicator, especially in desalination and separation processes. The overall recovery ratio is defined as a ratio of freshwater produced from each effect to the feed water applied to the V-MEMD unit:

(15) $$\%R=\frac{\sum_{i=2}^{N}{\dot{m}}_{D,i}}{{\dot{m}}_{F3}}\times 100$$

The concentration factor is one of the important indicators used to characterize the salt concentration within each effect during the evaporation process, which in turn negatively affects the performance of the membrane distillation layer. It is defined as a ratio of the effect’s salinity to the feed salinity, as shown as follows:

(16) $$C_{f,i}=\frac{C_{i}}{C_{F3}}$$

#### 4.2.2. Energy Indicators

There are also performance indicators that are directly related to the amount of energy (e.g., thermal energy and electrical energy), which are consumed by the desalination units. Among those indicators are the gain output ratio (GOR), heat recovery factor (Hr), specific thermal energy consumption (STEC), specific electric energy consumption (SEEC), and the effectiveness of the heat pump system (ε*_hp_*). They can be expressed as follows:

The gain output ratio (GOR) is a standard performance indicator for expressing the maximum thermal energy expansion through the cascade effects compared to the thermal energy applied to the V-MEMD unit. It can be determined from [10,24]:

(17) $$\mathrm{GOR}=\frac{\sum_{i=2}^{N}{\dot{m}}_{D,i}h_{Dfg,i}}{\emptyset_{H}}$$

The heat recovery factor in this study is defined as a ratio of the latent heat pushed to the next effect to the latent heat received from the previous effect. It can be expressed for each effect as follows:

(18) $${\mathrm{Hr}}_{i}=\frac{\emptyset_{l,i+1}}{\emptyset_{l,i}}$$

The specific thermal energy consumption (STEC) is the most critical performance indicator used in evaluating desalination systems. It is known as the amount of thermal energy consumed by the V-MEMD system to produce one cubic meter of distillate water; it can be expressed as follows:

(19) $$\mathrm{STEC}=\frac{\emptyset_{H}}{(\sum_{i=2}^{N}{\dot{m}}_{D,i})/\rho_{D,i}}$$

The specific electric energy consumption (SEEC) assesses how much electrical energy is required to operate the V-MEMD system in order to produce one cubic meter of distillate water. It can be expressed as follows:

(20) $$\mathrm{SEEC}=\frac{W_{p}+W_{R}}{(\sum_{i=2}^{N}{\dot{m}}_{D,i})/\rho_{D,i}}$$

The effectiveness of the heat pump system was also examined, and it can be expressed as follows:

(21) $$\varepsilon_{hp}=\frac{{\dot{m}}_{F2}C_{p}\left(T_{F3}-T_{F2}\right)}{{\dot{m}}_{F2}C_{p}\left(T_{hp}-T_{F2}\right)}$$

#### 4.2.3. Exergy Indicators

Using the performance indicators that are directly related to the exergy can help to realize the true depth of energy and understand the efficiency of the separation process. Therefore, among those indicators are the specific thermal exergy consumption (STXC), the exergy destruction (*Ψ_des_*), and exergetic efficiency (*η_ex_*). They can be expressed in the following order:

(22) $$\mathrm{STXC}=\frac{{\dot{m}}_{S2}\left(\varphi_{S2}-\varphi_{S3}\right)}{(\sum_{i=2}^{N}{\dot{m}}_{D,i})/\rho_{D,i}}$$

Exergy destruction is a valuable performance indicator for detecting energy loss through the cascade effects of the V-MEMD unit or the V-MEMD system components. The following expression will be used [24]:

(23) $$\%\Psi_{des,i}=\frac{\Psi_{des,i}}{\Psi_{des}}\times 100$$

The minimum work required to achieve the desired separation in the V-MEMD unit is defined as the difference between exergies of brine, distillate, and feed streams. It can be expressed as follows:

(24) $$W_{min}=\left({\dot{m}}_{b}\varphi_{b}\right)+\left(\overset{N}{\underset{i=2}{\sum }}{\dot{m}}_{D,i}\varphi_{D,i}\right)-\left({\dot{m}}_{F3}\varphi_{F3}\right)$$

In addition, the exergetic efficiency of the V-MEMD system is determined by the following formula:

(25) $$\%\eta_{ex}=\frac{W_{min}}{\emptyset_{H}\left[1-\frac{T_{i}}{T_{h,ave}}\right]+W_{R}+W_{p}}$$

## 5. Results and Discussion

### 5.1. Model Validation

**Figure 6:**
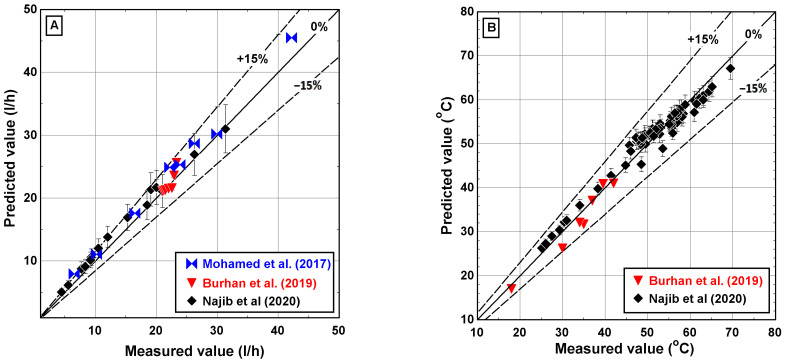
Comparison between experimental measurements and numerical predicted values of the mathematical model for (**A**) distillate water flow and (**B**) temperatures.

The experimental data generated from the V-MEME setup are used to validate the mathematical model. The validation results cover a wide range of operating conditions with maximum relative uncertainty for all variables, as specified in Table 3. BnR PLC was used to control the V-MEME unit and record process parameters except for the feed salinity. Feed salinity was measured manually using HANNA portable device. Figure 6 shows a comparison between experimental measurements and numerically predicted values of the mathematical model. Each data point denotes a unique operating condition specified in Table 3. Furthermore, the mathematical model results were also verified against Burhan’s results [27] and Mohamed’s results [9]. Generally, an excellent model-plant agreement is obtained. As shown in Figure 6A, the model-predicted distillate water production rate over the entire range of operating conditions is within a ±15 deviation band. The observed discrepancies may be attributed to three reasons: (i) knowledge of the mass and heat transfer processes in the spacer-filled channels, (ii) modeling assumptions, and (iii) fluctuation of recording parameters. As far as the predicted temperatures are concerned, they are in excellent agreement with the experimental measurements. The maximum model-plant discrepancy does not exceed the ±15 deviation band, as described in Figure 6B.

### 5.2. Estimating the Thermodynamic Properties

This section focuses on evaluating the thermodynamics properties of the V-MEMD unit under different feed water flow configurations (i.e., CFC and FFC), as described in Section 3. The feed salinity during this study was maintained constant at 1260 ppm (1.260 g/kg water). In addition, the thermodynamics properties of feed water were treated as a real mixture, as illustrated in [28]. The best-operating conditions were chosen based on conclusions from previous studies [9,10,11,13,14,15,18]. The cold water temperature (T*_C_*_1_) was maintained constant by the heat pump system at around 20 °C, and its flow (${\dot{v}}_{C1}$) was fixed at 405 L/h. Hot water flow (${\dot{v}}_{H1}$) and its temperature (T*_H_*_1_) were maintained at 900 L/h and 80 °C. Cold-side absolute pressure P*_v_* within effects was considered constant during the distillation process at 11.5 kPa. The dead state conditions have taken at T_o_ = 25 °C, P_o_ = 101.3 kPa, and C_o_ = 1260 ppm. The conservation equations are used to predict each point state in different feed water configurations. Table 4 shows a simple case study for different feed water configurations under specific input operating conditions. By inspecting the mass rate of port D5 in Table 4, the amount of vapor accumulated in the last effect (i.e., condenser compartment) of the V-MEMD unit for FFC is lower than that found in CFC, which makes the outlet temperature of the cold water stream (T*_C_*_2_) not exceed 25 °C. Therefore, the cold water stream loses its importance in preheating the feed water stream in HX-2. In addition, the heat required to be removed from the last effect by the heat pump system is low, and as a result, the heat rejected from the heat pump system cannot raise the feed water temperature to the desired temperature. A decrease in the feed water temperature means a decrease in the performance of the V-MEMD system, as described in [10,24,29]. Moreover, in CFC, the cold water stream carried high energy due to the increased amount of vapor accumulated in the last effect, so it played a positive role in the preheating of the feed water in HX-2. Moreover, the abundance of energy in the heat pump system, which is gained by removing heat from the last effect, played a positive role in raising the feed water temperature to 60 °C. However, the high heat removed also played a negative role manifested by increasing the electrical energy consumption.

### 5.3. Performance Evaluation

A comprehensive study on the two feed-flow configurations of the V-MEMD system (FFC and CFC) was applied under specific feed water conditions while the other operating conditions were kept constant. The FFC was experimentally and theoretically verified previously by Najib et al. [10,24]. The results showed that increasing the feed water flow at a low temperature had a negative impact on the performance of the V-MEMD system. Hence, this study aims to investigate the impact of the feed water conditions on the CFC and highlight the main differences between the two feed-flow configurations. The following section explains the effect of feed water conditions.

#### 5.3.1. Internal Performance Evaluation

**Figure 7:**
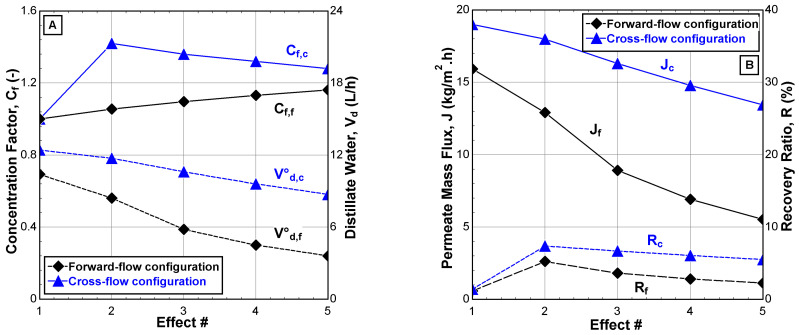
Internal performance indicators of the V-MEMD unit. (**A**) Concentration factor and distillate water, (**B**) permeate mass flux and recovery ratio (${\dot{v}}_{H1}$ = 900 L/h, $T_{H1}$ = 75 °C, ${\dot{v}}_{C1}$ = 405 L/h, $T_{C1}$ = 20 °C, ${\dot{v}}_{F3}$ = 159 L/h, $C_{F3}$ = 1260 ppm, and $P_{v}$ = 11.5 kPa).

It is important to understand the internal (effect-to-effect) performance behaviors of the V-MEMD unit. In order to realize the defects and obstacles that negatively affect the transfer of mass and heat through the cascaded effects. Figure 7 shows the variation of internal performance indicators related to productivity through the cascaded effects. The advantage of the CFC is clear in the distillate water, ${\dot{v}}_{d}$, permeate mass flux, J, and recovery ratio, R, that can reach 2–3 times that found in the FFC. Moreover, it is expected to increase the concentration factor, $C_{f}$, of the CFC, which reaches a maximum of 1.4 due to the increase in evaporation and a decrease in the feed water flow in the effect’s channel, as shown in Figure 7A. Moreover, it was observed that the highest and lowest results of internal performance indicators were recorded in the first effect; this is due to their reference to the hot water stream conditions (e.g., ${\dot{v}}_{H1}$ = 900 L/h, $T_{H1}$ = 75 °C, and $C_{H1}≅0\;\mathrm{ppm}$) in the evaporator compartment.

**Figure 8:**
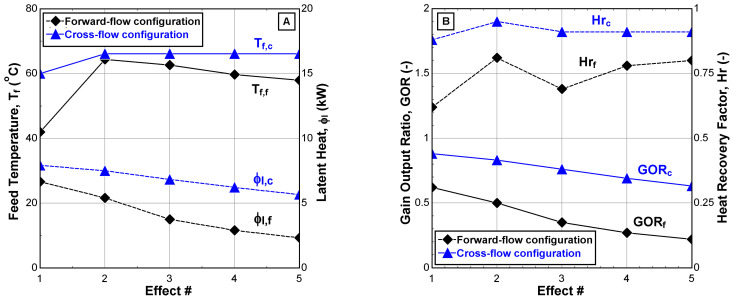
Internal performance indicators of the V-MEMD unit. (**A**) Feed water temperature and latent heat, (**B**) gain output ratio and heat recovery factor (${\dot{v}}_{H1}$ = 900 L/h, $T_{H1}$ = 75 °C, ${\dot{v}}_{C1}$ = 405 L/h, $T_{C1}$ = 20 °C, ${\dot{v}}_{F3}$ = 159 L/h, $C_{F3}$ = 1260 ppm, and $P_{v}$ = 11.5 kPa).

Figure 8 shows the variation of internal performance indicators related to thermal energy through the cascade effects. The advantage of the CFC in thermal energy came from feeding all effects with water solution having the same temperature (T*_F_*_4_~66.2 °C) as applied in conventional desalination systems [22]. This enhances the latent heat transfer through the cascade effects, as shown in Figure 8A, as well as the other performance indicators, as shown in Figure 8B. The average enhancement percentages of heat recovery factor, Hr, gain output ratio, and GOR relative to maximum values of FFC can reach up to 28% and 60%, respectively. However, the latent heat gradient is still observed through the cascade effects of both configurations but with different slopes; their reduction percentages can reach 9% for the CFC and 25% for the FFC.

**Figure 9:**
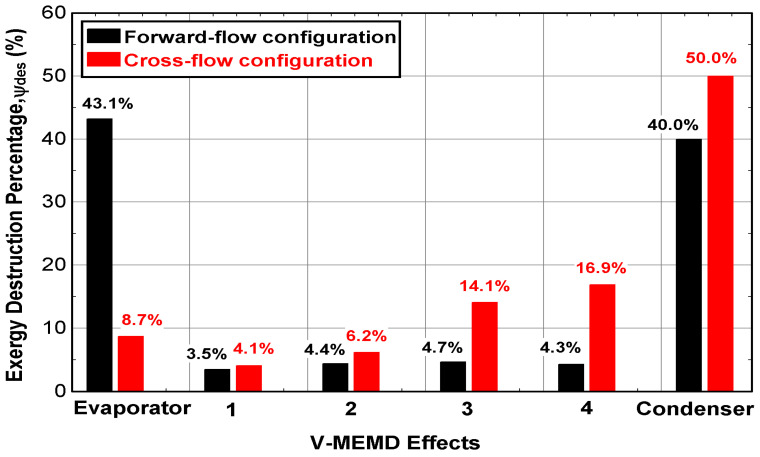
Exergy destruction percentage through the cascade effect of the V-MEMD unit (${\dot{v}}_{H1}$ = 900 L/h, $T_{H1}$ = 75 °C, ${\dot{v}}_{C1}$ = 405 L/h, $T_{C1}$ = 20 °C, ${\dot{v}}_{F3}$ = 159 L/h, $C_{F3}$ = 1260 ppm, and $P_{v}$ = 11.5 kPa).

As mentioned previously, increasing the feed water temperature played a critical role in reducing thermal energy consumption in the evaporator compartment [24]. As shown in Figure 9, the impact of CFC was evident in reducing the exergy destruction percentage in the evaporator compartment to 8.7% out of 43.1% in FFC due to the high inlet temperature of the feed water that reached up to 60 °C. No significant difference was recorded in the first to fourth effect when the FFC is enabled. In contrast, the exergy destruction percentage in the CFC increased exponentially through the cascade effects to reach the maximum value in the condenser. This may be attributed to the temperature distribution and energy dissipation through the cascade effects for two different feed-flow configurations.

#### 5.3.2. Global Performance Evaluation

**Figure 10:**
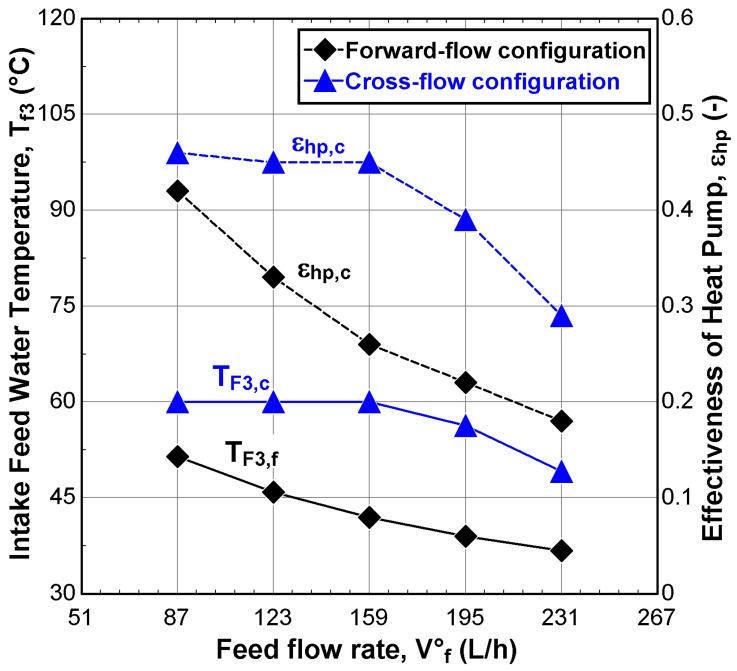
Effect of the feed water flow, ${\dot{v}}_{F3}$ on the feed water temperature and the effectiveness of the heat pump system (${\dot{v}}_{H1}$ = 900 L/h, $T_{H1}$ = 75 °C, ${\dot{v}}_{C1}$ = 405 L/h, $T_{C1}$ = 20 °C, $C_{F3}$ = 1260 ppm, and $P_{v}$ = 11.5 kPa).

It is equally important to understand the global performance behaviors of the V-MEMD unit with respect to its components, such as the heat pump system and heat exchangers. This will help identify the existence and the location of deficiencies throughout the entire system. Figure 10 shows the effect of the feed water flow on the intake feed water temperature (T*_F_*_3_) and the effectiveness of the heat pump system. The results showed, for both configurations, a decline in the feed water temperature and in the effectiveness of the heat pump system when the feed water flow increased from 87 L/h to 231 L/h. The reason is that the heat rejected from the heat pump system was not enough to raise the feed water temperature to the desired temperature. However, CFC provided more stable performance when the feed water flow was less than 159 L/h; this could be attributed to the abundance of heat in the heat pump system and the low thermal capacitance of the feed water.

**Figure 11:**
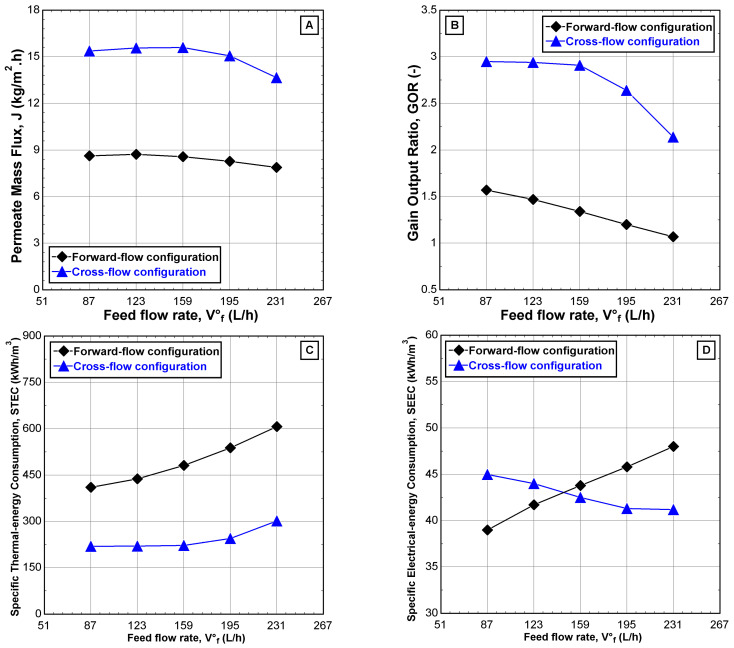
Effect of the feed water flow, ${\dot{v}}_{F3}$ on (**A**) permeate mass flux, (**B**) gain output ratio, (**C**) specific thermal energy consumption, and (**D**) specific electrical energy consumption for different feed-flow configurations (${\dot{v}}_{H1}$ = 900 L/h, $T_{H1}$ = 75 °C, ${\dot{v}}_{C1}$ = 405 L/h, $T_{C1}$ = 20 °C, $C_{F3}$ = 1260 ppm, and $P_{v}$ = 11.5 kPa).

According to the results obtained from Figure 10, increasing feed water flows negatively affected the global performance indicators for both configurations with some preference for CFC. The enhancements of T*_F_*_3_ and $\varepsilon_{hp}$ in CFC are probably up to twice as much as in FFC. Indeed, the behavior of the FFC was expected, as addressed by Najib et al. [10,24,29]. Figure 11A–C showed stability in the permeate mass flux, *J*, gain output ratio, GOR, and specific thermal energy consumption, STEC, for CFC when the feed water flow is less than 159 L/h; where their corresponding values can approach 15.5 kg/m^2^·h, 2.94, and 220 kWh/m^3^, respectively. The reason for this behavior is most likely to invest the heat rejected from the heat pump system into preheating the feed water. Interestingly, the performance indicator related to electrical energy (SEEC) is the best in FFC when the feed water is less than 159 L/h. Due to the low consumption of electrical work spent in the heat pump system to remove the heat accumulated in the last effect of the V-MEMD unit. On the contrary, SEEC exhibits better behavior in CFC when the feed water flow was greater than 159 L/h as a result of the low heat rejected from the heat pump system, as shown in Figure 11D.

**Figure 12:**
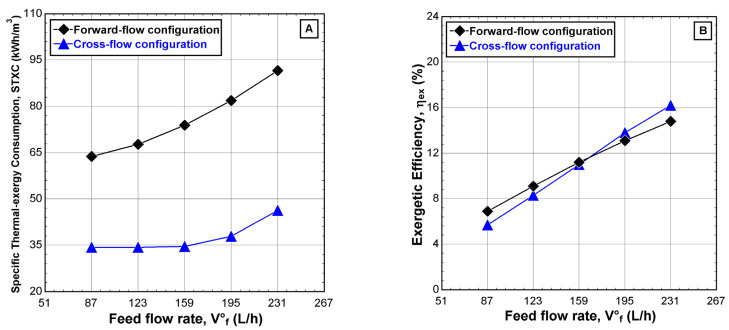
Effect of the feed water flow, ${\dot{v}}_{F3}$ on (**A**) specific thermal exergy consumption and (**B**) exergetic efficiency for different feed-flow configurations (${\dot{v}}_{H1}$ = 900 L/h, $T_{H1}$ = 75 °C, ${\dot{v}}_{C1}$ = 405 L/h, $T_{C1}$ = 20 °C, $C_{F3}$ = 1260 ppm, and $P_{v}$ = 11.5 kPa).

Figure 12 shows the variation of the specific thermal exergy consumption, STXC, and the exergetic efficiency, *η_ex_*, over the range of the feed water flow. The results showed similar behavior to that of the STEC (Figure 11C). The reason may be attributed to the fluctuation of the heat compensation in the evaporator and a decrease in productivity when the feed water increases from 87 L/h to 231 L/h, as shown in Figure 12A. The average enhancement percentage for STXC can reach around 50% when the CFC is enabled. The exergetic efficiency, *η_ex_*, increased linearly for both configurations over the range of the feed water flow. Moreover, no significant difference in the *η_ex_* was recorded between the two configurations, as shown in Figure 12B. The reason for the almost identical behavior of *η_ex_* for both configurations might be attributed to the unbalance between the heat compensation in the evaporator compartment and the electrical energy paid to the heat pump system to condensate the accumulated vapor in the condenser compartment.

**Figure 13:**
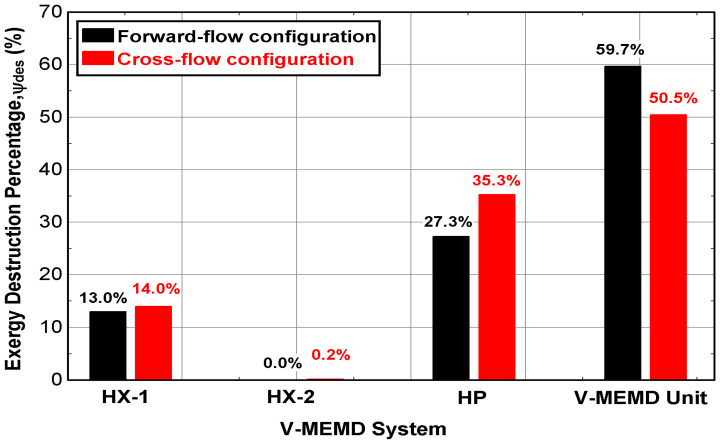
Exergy destruction percentage of the main components of the V-MEMD system (${\dot{v}}_{H1}$ = 900 L/h, $T_{H1}$ = 75 °C, ${\dot{v}}_{C1}$ = 405 L/h, $T_{C1}$ = 20 °C, ${\dot{v}}_{F3}$ = 159 L/h, $C_{F3}$ = 1260 ppm, and $P_{v}$ = 11.5 kPa).

Figure 13 demonstrates the exergy destruction in the major components of the V-MEMD system. Based on Table 4, the exergy destruction in the CFC ($\Psi_{des}$ = 2292 W) is greater than in the FFC ($\Psi_{des}$ = 1921 W); however, the difference in the percentage of the exergy destruction in the heat exchangers can be neglected. In contrast, the scenario is completely different for the heat pump system and the V-MEMD unit. In the heat pump system, its heat rejected was not fully invested in preheating the feed water temperature in the CFC, so the difference was estimated at 8%, as shown in Figure 13 for HP. Moreover, a positive achievement of the CFC in the exergy destruction in the V-MEMD unit. The exergy destruction percentage is reduced to 50.5% compared to 59.7% in the FFC. The reason is most likely due to the effectiveness of water separation between the two configurations.

Table 5 summarizes the main characteristics of the previous works [10,30], considering the desalination system using the V-MEMD process. The table shows a comparison with the present work. In the FFC, the ${\dot{v}}_{d}$, J, R, GOR, and STEC are within the range of Najib et al. [10] and Chen et al. [30] without any noticeable improvement in the V-MEMD system’s performance. The present investigation of the CFC showed enhancement of the performance indicators, especially the performance indicators associated with productivity (i.e., ${\dot{v}}_{d}$ and J). As far as *R* is concerned, it falls within the range of the work of Chen et al. [30]; this could be attributed to the low feed water flow (10 L/h to 50 L/h). Similarly, the STEC also falls within the range of the previous works [10,30]; their investigation covered a wide range of the hot water temperatures from 55 °C to 75 °C compared to our investigation, which was limited to 75 °C. Not surprisingly, the SEEC values of the present work are lower compared to Najib et al. [10] because Najib’s work included in their account the electric energy of all components of the solar-desalination system.

## 6. Conclusions

It is known that the CFC has an advantage when used in the conventional desalination systems due to avoiding the accumulation of salinity that adversely influences the desalination systems. Inspired by this advantage, the implementation of CFC in the V-MEMD unit is investigated. The study is based on the previously obtained best-operating conditions of the V-MEMD unit to compare the performance of operating under two different feed-flow configurations (i.e., CFC and FFC). Evidently, the CFC was found beneficial in improving the performance indicators of the V-MEMD unit. This is attained by improving the latent heat transfer through the cascade effects of several factors:

Evenly dividing the feed water stream to reduce the feed water flow in the channels so that the heat from the previous effect can be easily compensated;

The increase in the evaporation process in the cascade effects had a positive role in preheating the feed water stream.

Unfortunately, increasing the rate of the evaporation process when the feed water flow was less than 159 L/h played a negative role in increasing the electric energy. Performance indicators related to the exergy are also studied because they have a significant role in detecting destructive heat in the internal structure of the V-MEMD unit and its components. Apparently, there was a discrepancy in the exergy destruction percentages within the cascade effects of the V-MEMD unit. Overall, the CFC outperformed FFC with respect to exergy destruction with an estimated enhancement of about 9.2%. No significant difference in the exergy destruction percentages was recorded in the heat exchangers. Unfortunately, the exergy destruction percentage of the CFC in the heat pump system has increased over that of FFC; this could be attributed to the heat rejected is not fully invested. Moreover, the exergetic efficiency under specific operating conditions over the range of the feed water flow showed no significant differences between the two configurations.

## Figures and Tables

**Table 1:** Characteristics and specifications of the MD.

Character of Layer	Specification
Hydrophobic membrane material	Polytetrafluoroethylene (PTFE)
Foil material	Polypropylene (PP)
Dimension	335 mm × 475 mm
Effective area of one effect	0.64 m^2^
Membrane thickness	~0.2 mm
Porosity (ε)	75%
Tortuosity (1/ε)	~1.33
Mean pore size	~0.2 µm

**Table 2:** Abbreviations of the V-MEMD system.

Device	Symbol	Description
Pumps	P-1	Hot water pump
	P-2	Feed water pump
	P-3	Cold water pump
	P-4	Vacuum pump
	P-5	Heated water pump
Heat exchangers	HX-1	First heat exchanger
	HX-2	Second heat exchanger

**Table 3:** Operating conditions.

Operating Condition	Range	Max. Relative Uncertainty (%)
Hot water flow, ${\dot{v}}_{H1}$ (L/h)	574.8–902.4	±2.74
Hot water temperature, $T_{H1}$ (°C)	54.5–75.03	±1.27
Feed water flow, ${\dot{v}}_{F3}$ (L/h)	59.4–154.2	±3.05
Feed water temperature, $T_{F3}$ (°C)	24–59.4	±2.12
Cold water flow, ${\dot{v}}_{C1}$ (L/h)	249.6–863.4	±2.99
Cold water temperature, $T_{C1}$ (°C)	17.7–41	±3.69
Feed salinity, $C_{F3}$ (ppm)	1260	±2.00
Cold-side absolute pressue, $P_{v}$ (kPa)	9.8–20	±1.27

**Table 4:** Thermodynamics properties of all states in different feed water flow configurations.

State	FFC							CFC						
	T (°C)	P (kPa)	m (kg/s)	C (ppm)	h (kJ/kg)	s (kJ/kg·k)	φ (kJ/kg)	T (°C)	P (kPa)	m (kg/s)	C (ppm)	h (kJ/kg)	s (kJ/kg·k)	φ (kJ/kg)
S1	95	101.3	0.1417	0	398.0	1.25	29.9	95	101.3	0.1417	0	398.0	1.25	29.9
S2	95.3	217.2	0.1417	0	399.3	1.26	30.2	95.3	217.2	0.1417	0	399.3	1.26	30.2
S3	76.8	205.1	0.1417	0	321.5	1.03	17.1	77.9	205.1	0.1417	0	326.2	1.05	17.7
H1	75	46.1	0.2437	0	313.9	1.02	15.9	75	46.1	0.2437	0	313.9	1.02	15.9
H2	64.5	31.8	0.2409	0	269.8	0.89	10.2	66.2	32.4	0.2403	0	276.9	0.91	10.9
H3	64.5	31.8	0.2437	0	269.8	0.89	10.2	66.2	32.4	0.2403	0	276.9	0.91	10.9
F0	25	101.3	0.0438	1260	104.6	0.37	0	25	101.3	0.0438	1260	104.6	0.37	0
F1	25	155.1	0.0438	1260	104.6	0.37	0.05	25	155.1	0.0438	1260	104.6	0.37	0.05
F2	25	143.1	0.0438	1260	104.4	0.37	0.04	31	148.1	0.0438	1260	129.7	0.45	0.29
F3	41.9	131.1	0.0438	1260	175.4	0.6	1.9	60	131.1	0.0438	1260	250.7	0.83	8.0
F4	64.4	124.3	0.0438	1260	268.9	0.89	9.9	66.1	125.1	0.0438	1260	276.1	0.90	10.8
D1	64.4	24.5	0.0028	0	269.8	0.89	10.1	66.2	26.3	0.0034	0	276.9	0.91	10.6
D2	62.6	22.5	0.0023	0	262.1	0.86	9.2	63.9	23.8	0.0032	0	267.0	0.88	9.6
D3	59.7	19.7	0.0016	0	249.9	0.83	7.9	60	20.0	0.0029	0	250.7	0.83	7.8
D4	57.9	18.2	0.0012	0	242.7	0.81	7.1	56.4	16.8	0.0026	0	235.9	0.79	6.3
D5	56.6	17.1	0.001	0	237	0.79	6.6	53.2	14.5	0.0024	0	222.5	0.74	5.1
B1	56.6	101.3	0.0377	1464	236.5	0.79	6.4	58.3^a^	101.3	0.0327	1695.2 ^a^	243.6 ^a^	0.81 ^a^	7.2 ^a^
C1	20	23.6	0.1123	0	83.9	0.30	0.19	20	23.6	0.1123	0	83.9	0.30	0.19
C2	25	17.0	0.1123	0	104.5	0.37	0.005	32.1	17.0	0.1123	0	134.3	0.47	0.35
C3	25	12.6	0.1123	0	104.5	0.37	0	29.7	12.6	0.1123	0	124.6	0.43	0.15

^a^: average value for all brine branches.

**Table 5:** Comparison of the present results with those of [10,30].

Reference Number	Q. Chen et al. [30]	Najib et al. [10]	The Present Work	
Feed-flow configuration	Forward	Forward	Forward	Cross
Membrane area, A (m^2^)	3.84	5.12	5.12	5.12
Feed water type	Liquid desiccant (LiCl)	Brackish water	Brackish water	Brackish water
Hot water temperature, $T_{H1}$ (°C)	50–70	55–75	75	75
Hot-flow rate, ${\dot{v}}_{H1}$ (L/h)	N/A	840	900	900
Cold-side absolute pressue, $P_{v}$ (kPa)	3.17–15.76	7.54–30.11	11.5	11.5
Cold water temperature, $T_{C1}$ (°C)	20–30	25–30	20	20
Feed-flow rate, ${\dot{v}}_{F3}$ (L/h)	10–50	87–139	87–231	87–231
Water output, ${\dot{v}}_{d}$ (L/h)	0–25	0–31.8	20.2–22.4	35–40
Permeate mass flux, J (kg/m^2^·h)	0–13.1 ^b^	0–12.3	7.9–8.7	13.7–15.3
Recovery ratio, R (%)	0–60 ^b^	0–36.8	8.8–25.7	15.3–46
Gain output ratio, GOR (-)	N/A	0–4.24	1.5–2.5	2.8–3.9
Specific thermal energy consumption, STEC (kWh/m^3^)	208–3334 ^b^	151–675	410–606	219–302
Specific electrical energy consumption, SEEC (kWh/m^3^)	N/A	61–399	39–48	41–45

N/A: not available. ^b^ Values calculated from the reported data.

## Data Availability

Not applicable.

## Nomenclature

A	Membrane area: m^2^
C	Salinity, ppm
CFC	Cross-flow configuration
*C* *_f_*	Concentration factor
COP	Coefficient of performance
FFC	Forward-flow configuration
GOR	Gain output ratio
h	Enthalpy, kJ/kg
h*_fg_*	Vaporization enthalpy, kJ/kg
Hr	Heat recovery factor
*J*	Permeate flux, kg/m^2^·h
m°	Mass flow rate, kg/s
P	Pressure, kPa
$P_{v}$	Cold-side absolute pressure, kPa
R	Recovery ratio, %
s	Entropy, kJ/kg·k
SEEC	Specific electrical energy consumption, kWh/m^3^
STEC	Specific thermal energy consumption, kWh/m^3^
STXC	Specific thermal exergy consumption, kWh/m^3^
T	Temperature, °C
$W_{p}$	Total work consumed by the V-MEMD unit’s pumps, W
$W_{R}$	Work supplied on the heat pump, W
$W_{min}$	Minimum work, W
v	Volume flow rate, L/h
**Greek**	
*ρ*	Density, kg/m^3^
*η_ex_*	Exergetic efficiency, %
$\varepsilon_{hp}$	Effectiveness of the heat pump system
ε	Porosity, %
$\emptyset_{H}$	Heat supplied on the first heat exchanger, W
$\emptyset_{H,E}$	Heat supplied on the evaporator compartment, W
$\emptyset_{hp}$	Heat rejected from the heat pump, W
$\emptyset_{C}$	Cooling heat, W
$\emptyset_{loss}$	Heat loss, W
$\emptyset_{l}$	Latent heat, W
φ	Exergy flow, kJ/kg
$\Psi_{des}$	Exergy destruction, W
**Subscript**	
F	Feed water
H	Hot water
C	Cold water
D	Distillate water
B	Brine stream
hp	Heat pump
E	Evaporator
*i*	*i*th effects
